# High-velocity projectile impact induced 9R phase in ultrafine-grained aluminium

**DOI:** 10.1038/s41467-017-01729-4

**Published:** 2017-11-21

**Authors:** Sichuang Xue, Zhe Fan, Olawale B. Lawal, Ramathasan Thevamaran, Qiang Li, Yue Liu, K. Y. Yu, Jian Wang, Edwin L. Thomas, Haiyan Wang, Xinghang Zhang

**Affiliations:** 1 School of Materials Engineering, Purdue University, West Lafayette, IN 47907 USA; 2 Department of Materials Science and NanoEngineering, Rice University, Houston, TX 77005 USA; 3 Department of Engineering Physics, University of Wisconsin-Madison, Madison, WI 53706 USA; 4 State Key Lab of Metal Matrix Composites, School of Materials Science and Engineering, Shanghai Jiao Tong Univeristy, Shanghai, 200240 China; 5 Department of Materials Science and Engineering, China University of Petroleum, Beijing, 102249 China; 6 Mechanical and Materials Engineering, University of Nebraska-Lincoln, Lincoln, NE 68588 USA

## Abstract

Aluminium typically deforms via full dislocations due to its high stacking fault energy. Twinning in aluminium, although difficult, may occur at low temperature and high strain rate. However, the 9R phase rarely occurs in aluminium simply because of its giant stacking fault energy. Here, by using a laser-induced projectile impact testing technique, we discover a deformation-induced 9R phase with tens of nm in width in ultrafine-grained aluminium with an average grain size of 140 nm, as confirmed by extensive post-impact microscopy analyses. The stability of the 9R phase is related to the existence of sessile Frank loops. Molecular dynamics simulations reveal the formation mechanisms of the 9R phase in aluminium. This study sheds lights on a deformation mechanism in metals with high stacking fault energies.

## Abstract (web summary)

Deformation-induced defects with high formation energy are difficult to nucleate in aluminium. Here, the authors use a miniaturized projectile impact to nucleate the high stacking fault energy 9R phase in a thin film of ultrafine-grained aluminium, and show sessile Frank loops stabilize it.

## Introduction

Coarse-grained (CG) metals with face-centered-cubic (FCC) structure typically deform by glide of full dislocations^1^. When the stacking fault energy (SFE) is low, deformation of FCC metals is often accommodated by abundant partial dislocations^2^. Furthermore, prior studies show that grain size can tailor the nature of dislocations during deformation. For instance, ultrafine-grained (UFG, with grain sizes of 100–1000 nm) and nanocrystalline (NC, with grain sizes of 100 nm or less) FCC metals can deform via slip of partial dislocations or by twinning (consecutive emission of partial dislocations on adjacent {111} planes)^3, 4^, since the Frank–Read sources become more difficult to operate when the grain size is < 1 µm^5, 6^. But in FCC metals with high SFEs, deformation twinning is in general difficult^7, 8^ because full dislocations are the major carriers for plastic deformation^7, 8^. By using molecular dynamics (MD) simulations, Van Swygenhoven et al.^9^ have shown that deformation twinning tends not to occur in NC aluminium (Al) (with a grain size of 12 nm or less) with a high SFE, ~160 mJ/m^2^, because the high ratio $\gamma _{\rm sf}/\gamma _{\rm usf}$ ($\gamma _{\rm sf}$ SFE, $\gamma _{\rm usf}$ unstable SFE) facilitates the nucleation and emission of trailing partials, which eliminate SFs stemming from the glide of the leading partials. Yamakov et al.^10^ have theoretically predicted that deformation twinning can occur in NC Al with grain sizes of 45 and 70 nm. Experimental studies show that deformation twins indeed form in NC and CG Al at low temperature and high strain rate^11–13^, but with a very low probability^11–13^. This could be ascribed to the GB-mediated deformation mechanisms that reduce the probability of deformation twinning^14, 15^.

The 9R phase has been observed in FCC metals with low SFEs, such as Cu (45 mJ/m^2^)^16, 17^, Ag (22 mJ/m^2^)^18^ and Au (40 mJ/m^2^)^19^. The 9R structure is comprised of a stacking fault ribbon consisting of a repeating unit of 9 {111} atomic layers (6 stacking fault planes and 3 normal stacking planes). Thus, the 9R phase has a much higher formation energy than that of a twin (containing only two stacking fault planes). Although deformation twinning has been observed in high SFE metals under extreme deformation conditions, the formation of the 9R phase via plastic deformation has never been reported in pure Al because the 9R phase is difficult to nucleate and is highly unstable even if nucleated.

In this study, we investigate the deformation mechanisms of sputter-deposited UFG Al thin film (containing a certain fraction of growth twins^20^) subjected to high-velocity micro-projectile impacts by using a laser-induced projectile impact test (α-LIPIT) technique^21–23^. Extensive post-mortem transmission electron microscopy (TEM) studies reveal several tens of nm wide, 9R phase regions in the impacted UFG Al, as well as abundant dislocation networks, along with grain rotation and fragmentation. A mechanism for the formation of 9R phase has been discovered in Al by using MD simulations. To accommodate the plastic deformation under high strain rate, the formation of 9R phase via dissociations of incoherent twin boundaries (ITBs) can occur even if there is a high-energy barrier. Frank loops play an important role to stabilize the 9R phase in Al.

## Results

### Microstructure characterization

**Fig. 1:**
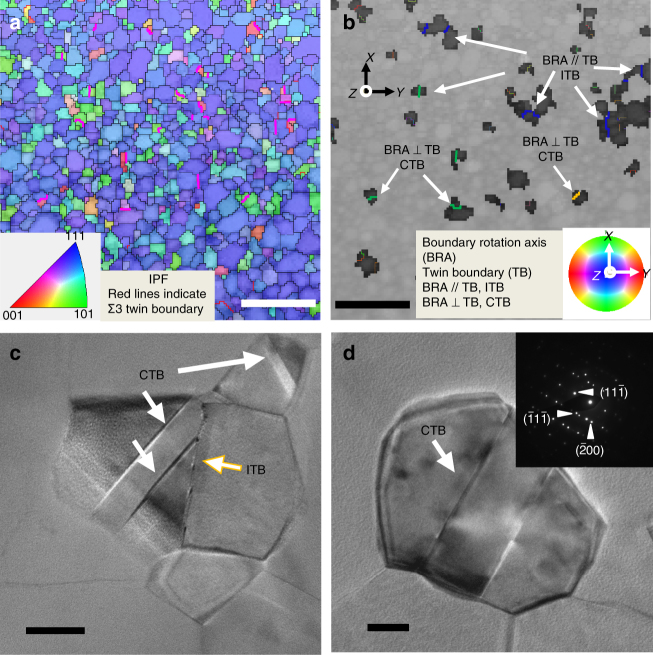
Microstructures of as-deposited ultrafine grained (UFG) Al thin film. **a** EBSD micrograph showing orientation map along the sample surface normal direction; the red lines indicate ∑3 twin boundaries. Scale bar, 1 µm. **b** The boundary rotation axis (BRA) map reveals the incoherent twin boundary (ITB) (when BRA//TB) and coherent twin boundary (CTB) (when BRA⊥TB). Scale bar, 1 µm. **c**, **d** Plan-view TEM images showing growth twins in as-deposited UFG Al thin films (inset of **d** shows the selected area diffraction (SAD) pattern of a grain containing growth twins). Scale bar, 20 nm

**Fig. 2:**
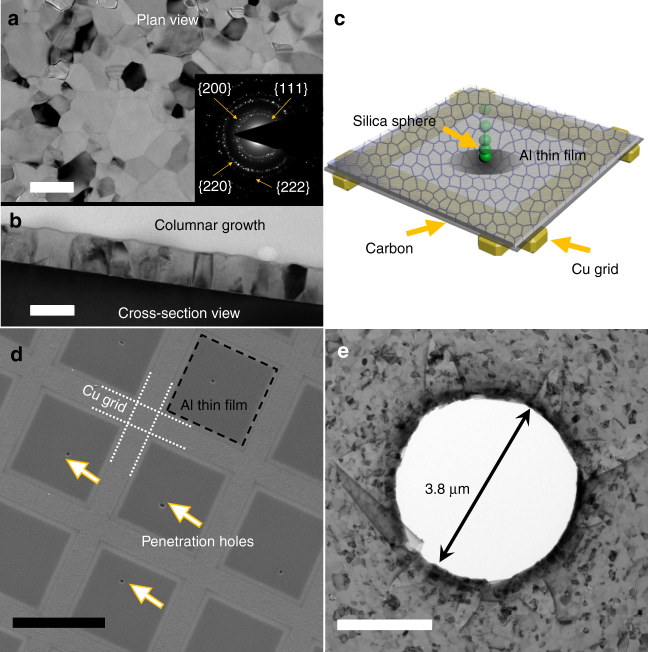
A laser-induced projectile impact testing (α-LIPIT) for UFG Al films with a thickness of 140 nm. **a** A plan-view TEM micrograph showing the as-deposited UFG Al film. Scale bar, 200 nm. **b** The cross-sectional view of the as-deposited Al thin film showing columnar grains. Scale bar, 100 nm. **c** A schematic of the α-LIPIT experiment. **d** A low magnification SEM micrograph showing the perforations induced by micro-projectiles in the UFG Al film supported by Cu TEM grid. Scale bar, 100 µm. **e** A representative TEM image showing the film morphology around a circular perforation. Scale bar, 2 µm

The electron backscatter diffraction (EBSD) micrograph and the corresponding orientation mapping analysis (Fig. 1) show that the as-deposited films have ultrafine grains with high-angle grain boundaries (GBs). The grain size of the as-deposited Al films (Supplementary Fig. 1) varies from 60 to 350 nm, with an average of ~140 nm. The red lines in Fig. 1a indicate the ∑3 {111} twin boundaries (TBs) in the as-deposited films. In order to differentiate the ∑3 coherent TBs (CTBs) and and ITBs, we need to examine the boundary rotation axis (BRA). As shown in Fig. 1b, the BRA is parallel to the ITB, but perpendicular to the CTB. TEM micrographs (Fig. 1c, d) and the inserted selected area diffraction (SAD) patterns confirm the formation of CTBs in the as-deposited UFG Al film. A plan-view TEM micrograph (Fig. 2a) and the inserted SAD patterns show the polycrystalline nature of the UFG Al film. A cross-sectional TEM micrograph shows that the grains are columnar (Fig. 2b).

As shown schematically in Fig. 2c, individual SiO_2_ microspheres (3.7 μm in diameter) were launched to impact the free-standing UFG Al films deposited on TEM grids. The projectile velocity was 600 m/s corresponding to strain rates of ~10^7^–10^8^/s. A plan-view scanning electron microscopy (SEM) micrograph of the impacted film is shown in Fig. 2d. Most of the perforated holes have a circular shape with a diameter similar to that of the SiO_2_ projectiles (Supplementary Fig. 2). The TEM micrograph in Fig. 2e shows a representative circular hole after micro-projectile penetration. TEM studies show that a majority of the impacted zones have limited number of radial cracks surrounding the perforated holes (Supplementary Fig. 3).

### Formation of the 9R phase

**Fig. 3:**
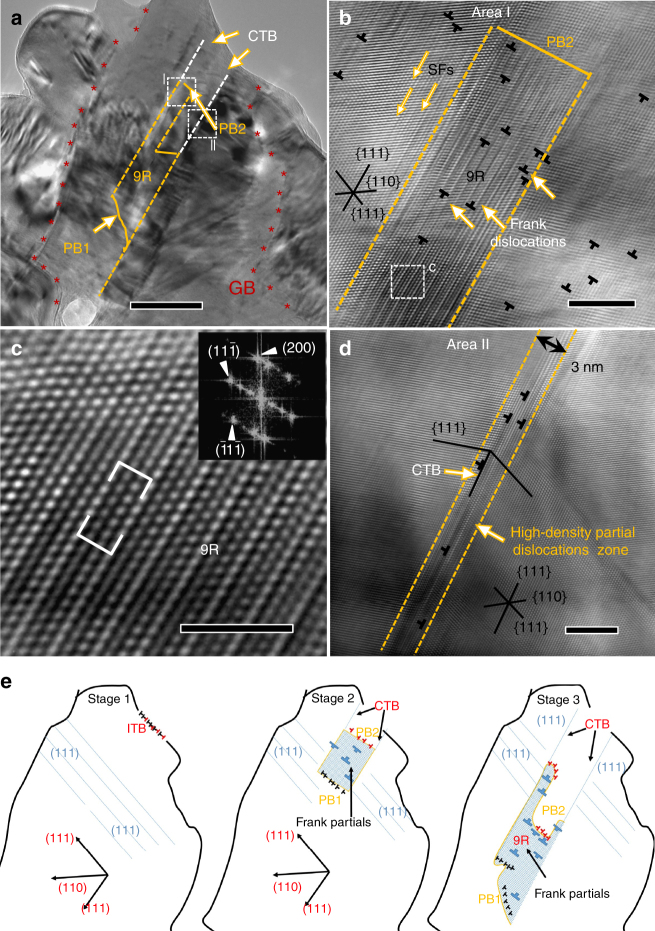
Projectile impact induced 9R phase in UFG Al films. **a** Overview of the microstructures of UFG Al adjacent to the edge of the perforated hole. CTBs bounding a giant 9R phase are identified. The phase boundaries (PB1 and PB2) separating the 9R phase from the matrix are also labeled. Two white boxes outline the defects shown at higher magnification in **c**, **d**. Red asterisks outline a grain boundary (GB). Scale bar, 50 nm. **b** The HRTEM micrograph reveals the formation of the giant 9R phase in UFG Al after impact. Numerous Frank partials are also observed in the 9R phase. Scale bar, 5 nm. **c** The HRTEM micrograph of the CTB from area 2. High-density Shockley partials are identified along the CTB. Scale bar, 2 nm. **d** HRTEM micrograph of the white box in **b** showing the 9R phase. Scale bar, 5 nm. **e** A schematic shows the deformation-induced 9R phase and the TBs. A section of the GB has the nature of an ITB with one Shockley partial on each adjacent {111} plane. High-strain-rate impact triggers the migration of partials along the ITB. The giant 9R region is over 100 nm long and is bounded between PB1 and PB2. Sessile Frank partials also form within the 9R phase and pin the trailing partials, stabilizing the 9R phase after the high-strain-rate impact.

A striking phenomenon is the formation of 9R phase regions in highly deformed areas near the edge of the perforation. Figure 3a shows a typical deformation twin formed near the edge of a perforation. The white dashed lines indicate two parallel CTBs. At the end of the two CTBs, a dashed orange line marks the boundary of an 80 nm wide 9R phase. The curved phase boundaries (PBs) separating the 9R phase from matrix are marked as PB1 and PB2. The two PBs associated with the 9R region are comprised of arrays of Shockley partial dislocations^24^. Specifically there is an array of edge type of Shockley partial dislocations located on one side of the 9R phase (forming PB1), and an array of mixed Shockley partial dislocations located on the opposite side of the 9R phase (forming PB2) (Supplementary Fig. 4). The two sets of partial dislocations on the PBs attract each other as the edge components of the two sets of partial dislocations have opposite signs.

One section of the 9R phase near the upper TB is analyzed by high-resolution TEM (HRTEM). The magnified view of the 9R phase in Fig. 3c confirms the periodic stacking sequence typically observed in the 9R phase^17^. As shown in Fig. 3b, near the left side of the giant 9R phase, multiple SFs are observed. Besides SFs, numerous Frank partial dislocations were also identified within the 9R phase. The magnified view of box 2 (in Fig. 3d) shows the deformation-induced CTB containing numerous Shockley partials. The schematics illustrate the formation of the 9R phase and CTBs in Fig. 3e, which will be discussed later in the paper.

### Grain fragmentation

**Fig. 4:**
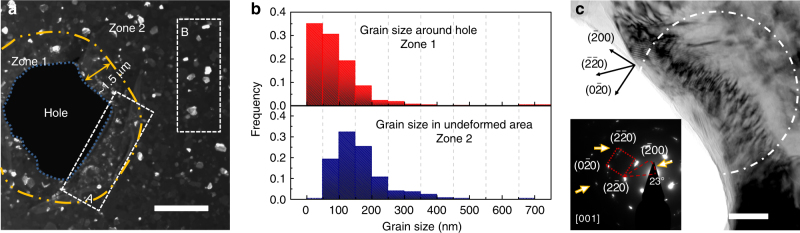
Grain fragmentation and coarsening near the perforations. **a** A dark-field TEM image of the grains near a perforation. The grains in area A in zone 1 (within the 1.5 μm wide impact zone) are typically smaller than those in area B in zone 2 (further away from the impact zone), indicating grain fragmentation during projectile impact. The yellow dash-dot line delineates the boundary between zone 1 (highly impacted zone) and zone 2 (less deformed area). Scale bar, 2 µm. **b** The statistical distribution of grain sizes in zone 1 and 2. **c** A low magnification bright field TEM micrograph showing the morphology of an elongated grain containing a high density of dislocations adjacent to a hole. The length of the grain is ~300 nm, and its width is ~70 nm. The inserted diffraction pattern shows the slightly axisymmetric elongated {220} diffraction spots examined along <100> diffraction zone axis, indicating subgrain rotation within the grain. Scale bar, 50 nm

In addition to the discovery of the impact-induced 9R phase, grain fragmentation was also frequently observed. A dark-field TEM micrograph in Fig. 4a shows the microstructure of the impacted films can be separated into two different zones based on the morphology of grains: a highly deformed zone 1 adjacent to the edge of the circular perforation, with a width of ~1.5 µm, and a less deformed annular zone 2 outside zone 1. Most grains in zone 2 have well-defined GBs and relatively uniform contrast, while inside zone 1, especially nearest the edge of the perforation, much smaller grains with irregular GBs are frequently observed. Comparison of the grain size distributions in the two zones (Fig. 4b) shows that the average grain size is reduced in zone 1 near perforated holes (Supplementary Fig. 5). Although grain fragmentation dominates the evolution of grain morphology inside the highly deformed area, grain coarsening has also been observed occasionally. As shown in Fig. 4c, a large elongated grain containing a large number of dislocations was observed near the edge of a different perforation. The inserted SAD pattern from the area marked by the white dash-dot line shows the single-crystal like diffraction pattern captured along the [001] zone axis. The large misorientation angle (> 20°) in the stretched {220} diffraction spots indicates grain rotation during the formation of the large grain.

### Dislocation network

**Fig. 5:**
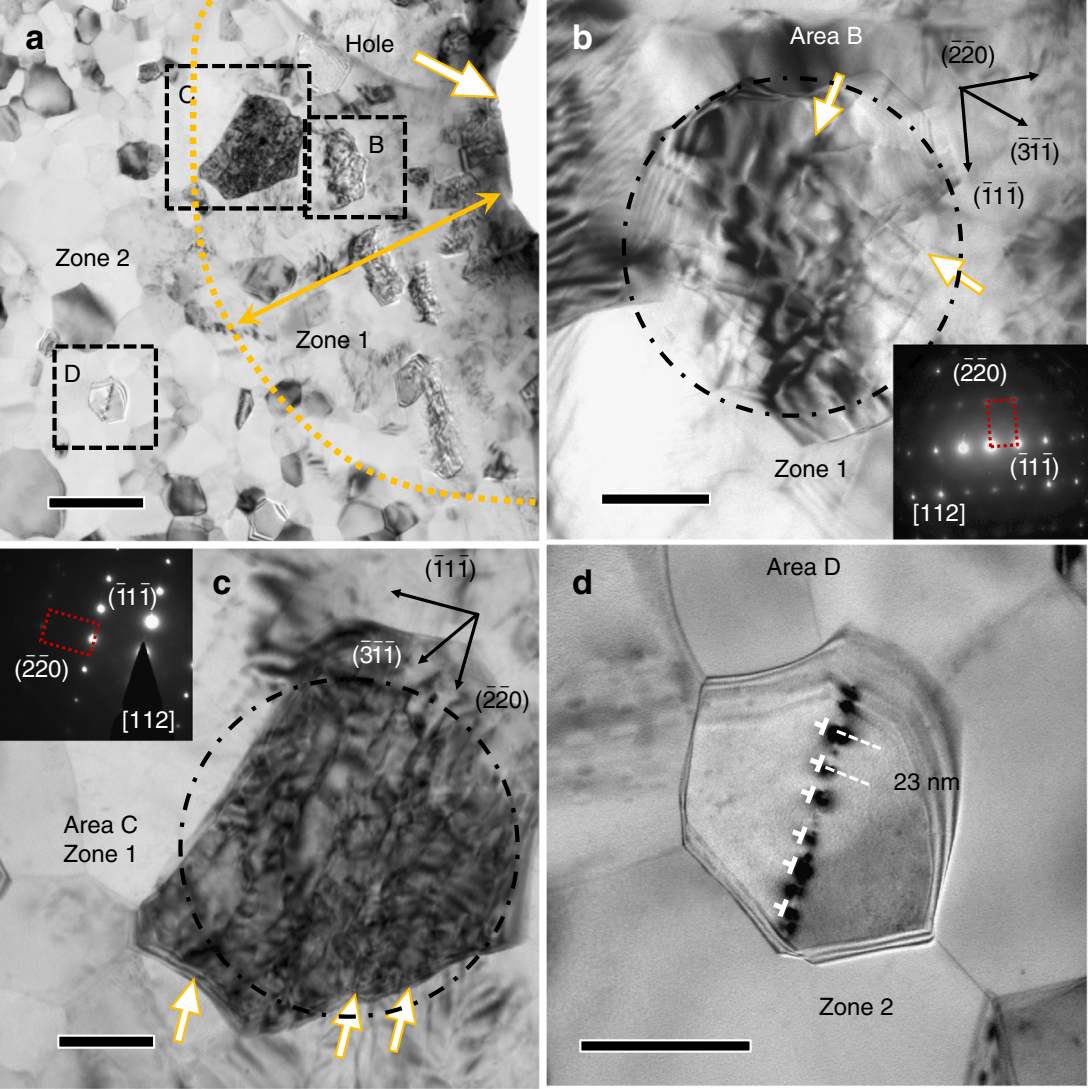
Dislocation networks after ballistic impact in bright field TEM images. **a** Low magnification micrograph showing the alteration of the microstructure near a perforated hole in UFG Al. Grains in zone 1 have greater defect density than those in zone 2. Scale bar, 500 nm. **b**, **c** Higher magnification views of dislocation networks in areas B and C. The white arrows indicate the traces of dislocations in large grains examined along the <112> zone axis. Scale bar, 100 nm. **d** High magnification image of the area D in zone 2 showing the edge-on view of an array of dislocations aligned along a CTB. A linear array of dislocation cores, separated by ~20 nm, is identified. Scale bar, 100 nm

Furthermore, a large number of dislocations induced by the high-strain-rate impact were observed in the deformed grains, and the defect density varies inversely with the relative distance of the grains to the perforated holes. In general, a large number of dislocations are frequently observed in grains located within zone 1 as shown in Fig. 5a. Moreover, many grains in zone 1 have irregular shapes and complex stress contours. Figure 5b shows tangled dislocations aligned nearly orthogonal to one another in a large grain in area B of zone 1. Another adjacent large grain in area C of zone 1 contains prominent parallel dislocation networks (Fig. 5c). TEM tilting experiments were performed to examine dislocations in these deformed grains (Supplementary Fig. 6). Although grains in zone 2 typically have less internal defects, arrays of dislocations are frequently observed in grains containing CTBs (growth twins). Figure 5d shows the enlarged image of the box D in zone 2, where an array of black dots was observed running straight across the entire grain. The black dots arise from the edge-on view of dislocation cores, and the straight line is a CTB of a growth twin inside the grain. Similar arrays of dislocations have been frequently observed in numerous other grains in zone 2 containing growth twins (Supplementary Fig. 7).

## Discussion

In FCC metals with low SFE, the core of a 1/2 <110> full dislocation can dissociate into two Shockley partial dislocations connected by a SF, which is also referred to as an extended dislocation^1, 25^. Abundant deformation twins or SFs have been observed in deformed FCC metals with low SFEs^4, 26–32^. In contrast, plastic deformation of monolithic CG Al typically does not lead to deformation twins or SFs due to the high SFE of aluminium^33, 34^. However, there is increasing evidence of deformation twinning in NC Al both experimentally and computationally^11–13, 35–37^. MD simulations^35^ reveal several twinning mechanisms in NC Al, including the overlap of two extended dislocations on adjacent slip planes; successive emission of multiple Shockley partial dislocations from GBs; and GB splitting and migration induced formation of TBs. Zhu et al.^4^ reviewed various twin formation scenarios related to the successive slip of partial dislocations in NC Al, Ni, and Cu. Briefly there are two partial dislocation emission processes, including monotonic activation of partial dislocations (MAPs) with the same Burgers vector and random activation of partial dislocations (RAPs)^38^. Wu et al.^38^ hypothesize that RAP is more favorable than MAP twinning mechanism since RAP induces little macroscopic strain due to zero net macroscopic strain, alleviating the strain on neighboring grains. Distinctive from the MAP and RAP deformation twinning mechanisms, a growth-induced 9R phase with zero net shear strain has been observed in FCC metals with low SFE^39–42^ and in CuZn alloys^43^. However, instead of the MAP and RAP mechanisms, the new deformation-induced 9R phase results from the consecutive slip of three partial dislocations on adjacent slip planes in a periodic manner. In Ag, the nucleation and migration of a ∑3{112} ITBs facilitate the formation of the 9R phase^24^. However, deformation twinning in Al remains a difficult, high-energy process as shown by the spontaneous detwinning during in situ TEM tensile experiments^44, 45^. Also, a deformation-induced 9R phase in Al has, to our knowledge, not yet been previously reported. Hence the formation of 80 nm wide 9R phase regions in UFG Al is unexpected. The following section will discuss the detailed formation mechanism of deformation-induced 9R phase in Al as revealed by MD simulations.

The non-equilibrium MD (NEMD) method has been used for the investigation of shockwave response of solids, because the large-scale MD simulations can generate steady plastic (or split elastic–plastic) waves. Prior MD simulations and experimental studies on shock response of single crystals and polycrystalline materials have shown qualitative and, in some cases, quantitative agreement^46–51^. To investigate the mechanisms for the formation of 9R phase in Al, we simulated the shock response of nanoscale columnar grains in Al using the plate spallation experiment^46, 52^. The stress state in surrounding zones around the perforated holes is quite complex during the penetration of a projectile through the film. As a projectile impacts, the film is stretched, resulting in an axisymmetric tensile stress. The tensile stresses generate resolved shear stresses acting on partial dislocations in ITBs. It is however very challenging to generate a tensile shock wave in MD simulations. We thus simulated the formation of the 9R phase in UFG Al with a compressive shock wave, because an effective resolved shear stress is naturally responsible for the gliding of partial dislocations.

**Fig. 6:**
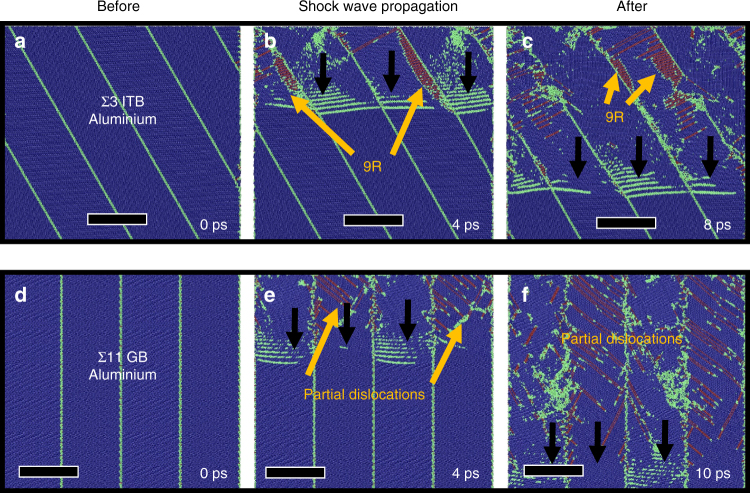
Molecular dynamics simulation of the shock-induced activity of partials in ∑11 GB and ∑3 ITB. **a**–**c** Snapshots of shock-induced dissociation of partial dislocations from compacted ∑3 {112} ITB in Al. **a** The ∑3 ITB with 15 nm domain size in Al film. Scale bar here and in subsequent panels is 15 nm. **b**, **c** When the shock wave passes through the ITBs, the 9R phase forms due to the dissociation of ITBs by the emission of partial dislocations from the ITBs. The red area bounded by the green lines is the 9R phase. **d**–**f** The snapshots of shock-induced nanotwin and partials (nucleation and emission of partial dislocations from ∑11 GB). **d** The ∑11 GB with 15 nm domain size in Al film. The GB contains Shockley partial dislocations every seven {111} atomic planes. **e**, **f** Partial dislocations are emitted from the GBs due to the shock wave

To inspect the influence of GB structures on the formation of 9R phases, we examined the shock response of NC Al with two different types of GBs under shock at a speed of 1 km/s. In the first case, NC Al contains four grains that have twin relation and form ∑3 {112} ITBs. The columnar grain size is 15 nm and the height is 75 nm. Topological analysis and microscopic characterization show that ∑3 {112} ITBs in FCC metals consist of a repeatable pattern involving three Shockley partial dislocations as one unit in three adjacent {111} atomic planes (Supplementary Fig. 8(a1–a3))^17, 29^. In comparison, we also shocked the columnar NC Al that contains ∑11 asymmetrical tilt GBs. The GB plane is parallel to ($\bar 252$) and ($\bar 414$) in the neighboring grains. The GB contains Shockley partial dislocations every seven {111} atomic planes (Supplementary Fig. 8 (b1, b2))^53^. Partial dislocations were nucleated at GBs and emitted into grains in two simulations (Fig. 6). Most importantly, the 9R phase is only observed in the NC Al containing ∑3 {112} ITBs (Fig. 6c). The formation of the 9R phase is ascribed to the emission of pre-existing Shockley partial dislocations. Under high shear stresses, it is expected that one set of partial dislocations on PB1 (Supplementary Fig. 4) in Al can glide along one direction away from the compacted ∑3 {112} and the rest of partial dislocations that have screw components with opposite signs on PB2 may glide towards the opposite direction if the gliding force acting on partials exceeds the Peierls barrier^24^. As a result, the 9R phase can propagate.

Several factors may contribute to the stabilization of the 9R phase induced by the high-strain-rate deformation. The high-resolution microscopy (Fig. 3b) shows the formation of numerous Frank partials inside the 9R phase. The Frank partials may arise from the interactions among high-density Shockley partials that are activated on different slip systems during projectile impact. These sessile Frank partials may act as barriers to block partials aligned on PB2 and thus enhance the stability of the 9R phase.

Besides the pinning effect from the sessile dislocations, the interaction force between the partials of b_1_ and partials of b_2_ and b_3_ (Supplementary Fig. 4) also plays a role in stabilizing the 9R phase. Different from the conventional repulsion between a leading partial and trailing partial dislocation bounding a stacking fault ribbon, the ∑3 (112) ITB consists of a periodic array of three different partial dislocations, b_1_, b_2_, and b_3_ (Supplementary Fig. 4a). These three partials have a net zero Burgers vector^17, 29^. As shown in Fig. 3e, at stage 1, a portion of the GB consists of ∑3{112} ITBs, which are confirmed by HRTEM images and EBSD data in Fig. 1a, b. Under high-strain-rate impact, one set of partial dislocations b_1_ on PB1 (as shown in Supplementary Fig. 4b) and the other set of partial dislocations (b_2_ and b_3_ on PB2) glide on {111} planes towards the opposite direction because the edge component of their Burgers vectors has an opposite sign. Consequently a 9R phase forms bounded by PB1 and PB2 (Supplementary Fig. 4c). However, PB2 will experience a high friction force due to its two-atomic layer core of the b_2_ and b_3_. Thus PB1 moves faster than PB2. As a consequence, PB2 will move towards PB1 in order to minimize the system energy due to the formation of the 9R phase, as shown in stage 2 in Fig. 3e ^29^. Finally, the partials in PB1 stop when the shock-induced stress is insufficient to drive their further migration (stage 3). Meanwhile, with the increasing separation distance, the attraction force between partials dislocations on PB1 and PB2 also quickly decreases and becomes insufficient to go over the barrier stress resulting from Peierls friction stress or the Frank partials, leading to the stable pinning of the 9R phase in the impacted UFG Al.

For a thin film specimen, free surfaces play different roles on the formation and annihilation of the 9R phase in our Al thin film. As the projectile penetrates through the Al film, the high shear stresses trigger the glide of dislocations. Once a dislocation glides away from the ITB into the grain, the image force due to free surfaces attracts the dislocation. For full dislocations, this attraction process reduces the dislocation density in the grains. While for partial dislocations, this attraction process facilitates the formation of the broad 9R phase. However, the 9R phase is a high-energy structure and can be considered to some extent as a high-density stacking fault. Hence after the projectile impact, free surfaces act as sources to nucleate partial dislocations that may annihilate 9R phase regions to reduce the system energy. As a consequence of the nearby free surfaces, less 9R phase regions will exist inside the thin film. The influence of the free surfaces might also explain why certain grains in the impacted zone do not appear to have a large number of dislocations. Although TBs are rare in monolithic CG Al prepared by rolling and annealing, numerous studies have shown that deformation twins can be introduced into NC Al^11, 13, 54, 55^. It is likely that high-strain-rate deformation may also introduce the 9R phase in NC Al containing deformation twins. Such a hypothesis requires further validation by experimental investigations.

In addition to the observation of deformation twins and the 9R phase near the perforations, dislocation slips and the formation of dislocation networks during projectile impact are ubiquitous during the plastic deformation of UFG Al^12^. In the highly deformed regions, the dislocation density can reach the order of 10^12^/cm^2^, comparable to that in heavily cold worked metals. The dislocation networks manifested by the complex stress contours accommodate the plastic deformation and facilitate energy dissipation during projectile impact. The impact induced grain fragmentation could be related to the formation of dislocation cell walls in the grains or significant shear induced grain rotation and refinement. Our recent study also shows that the as-deposited UFG Al films contain a certain fraction of growth twins^20^. These growth twins interact with the impact induced dislocations and act as pinning centers to store dislocations (Fig. 5d).

In summary, broad 9R phase regions have been discovered in UFG Al subjected to high-velocity projectile impact in spite of the characteristic high SFE of Al. The 9R phase arises from shock-induced rapid migration of partials from ITB seeds in as-deposited Al. A deformation twinning mechanism has been discovered in a FCC metal with a high SFE. The stability of the giant 9R phase is due to the pinning of partial dislocations by abundant sessile dislocations within the 9R phase. The methodology of using a novel micro-projectile impact technique on TEM specimens opens a new avenue for high-throughput examination of high-strain-rate impact induced damage and plasticity in a broad range of metallic materials.

## Methods

### Sample preparation and projectile impact experiments

Al thin films, ~140 nm in thickness, were sputter-deposited onto carbon film (25 nm thick)-coated copper TEM grids. The base pressure of the vacuum chamber was ~8 × 10^−8^ torr. The Transmission Kikuchi Diffraction (also referred to as t-EBSD) technique was used to collect crystallographic information on the Al thin films in the transmission mode of a Tescan FERA-3 scanning electron microscope operated at 28 kV. The EBSD data analyses were performed using the Channel 5 software suite. The scan area was 4.6 × 4.6 µm with a step size of 20 nm. To explore the deformation mechanisms of UFG Al at high strain rates, we used a recently developed LIPIT technique, where high-velocity monodispersed silica microspheres (~3.7 µm in diameter) impact and penetrate the UFG Al film. Individual silica microspheres were launched using a laser pulse, towards Al films at high velocities (~ 600 m/s) to generate high-strain-rate (~10^7^–10^8^/s) deformation in a local region. Approximately a dozen impact experiments using silica micro-projectiles were performed at different grid areas (i.e., each time the projectile impacts a pristine portion of the UFG Al films). Free-standing films were used to avoid the complexity of back-stress waves that are typically seen in mechanically clamped bulk shock-loaded specimens.

### Microstructure characterization

After the projectile penetration experiments, the shape of the perforated holes in UFG Al film was examined by using an FEI Quanta 600 scanning electron microscope operated at 10 kV. TEM analysis was performed on an FEI Tecnai F20 ST microscope operated at 200 kV to characterize the evolution of the microstructure near the impacted regions. To further probe the impact-induced microstructural changes in the UFG Al films, HRTEM experiments were performed.

### MD simulations

Al specimens were relaxed by energy minimization using the quenching MD method, followed by equilibration using isothermal isobaric ensemble at 300 K and 0 GPa pressure for 50 ps. The time-step in NEMD simulations was chosen to be 0.2 fs to ensure numerical stability. The velocity-Verlet algorithm was adopted to solve the MD equations. The computational model for the plate impact experiment comprises two parts using the same material. One part is fixed (representing the impactor) and the other part is the target, i.e., the UFG Al sample. Two-dimensional periodic boundary conditions were used in the perpendicular directions. The target was shocked by ramming it at a speed of 1 km/s against the fixed impactor. The atomic interactions in Al were described by an accurate embedded atom method (EAM) potential developed by Winey et al.^52^. The validity of the EAM potential under strong shock conditions was confirmed by comparing Hugoniot curves (P–V and P–T curves) and the melting curve with experimental data^52^.

### Data availability

The data that support the findings of this study are available from the corresponding authors upon reasonable request.

## Electronic supplementary material

Supplementary Information

Description of Additional Supplementary Files

Supplementary Movie 1

Supplementary Movie 2
