# Supplementary material for: High-velocity projectile impact induced 9R phase in ultrafine-grained aluminium
Source: Nat Commun. 2017 Nov 21;8:1653. doi: 10.1038/s41467-017-01729-4 (PMC5698461; doi:10.1038/s41467-017-01729-4)
Supplement: Supplementary file 1 — Supplementary Information [file 41467_2017_1729_MOESM1_ESM.pdf]

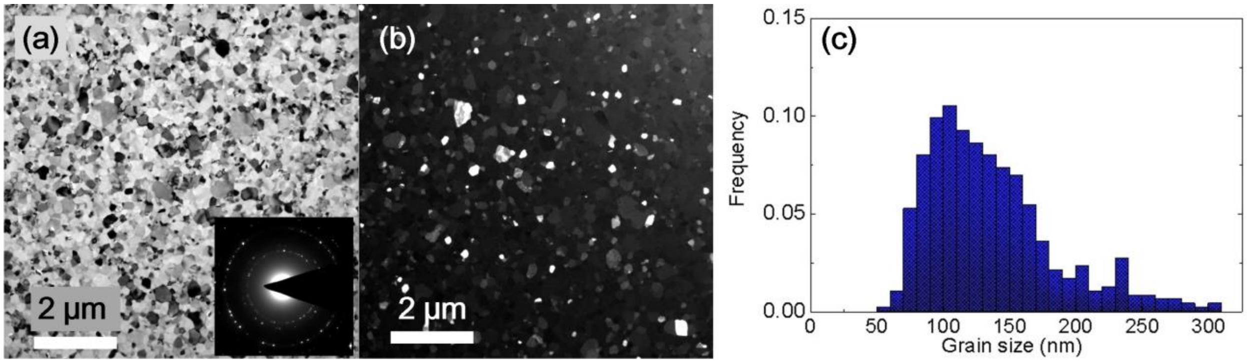

Supplementary Figure 1. Microstructure of the as-deposited polycrystalline UFG Al thin film. (a) A bright-field TEM image and inserted selected area diffraction (SAD) pattern showing the formation of high angle grain boundaries and ultra-fine grains. (b) A dark-field TEM micrograph showing the formation of UFGs. (c) The statistical grain size distribution showing an average grain size of 140 nm in the as-deposited film.

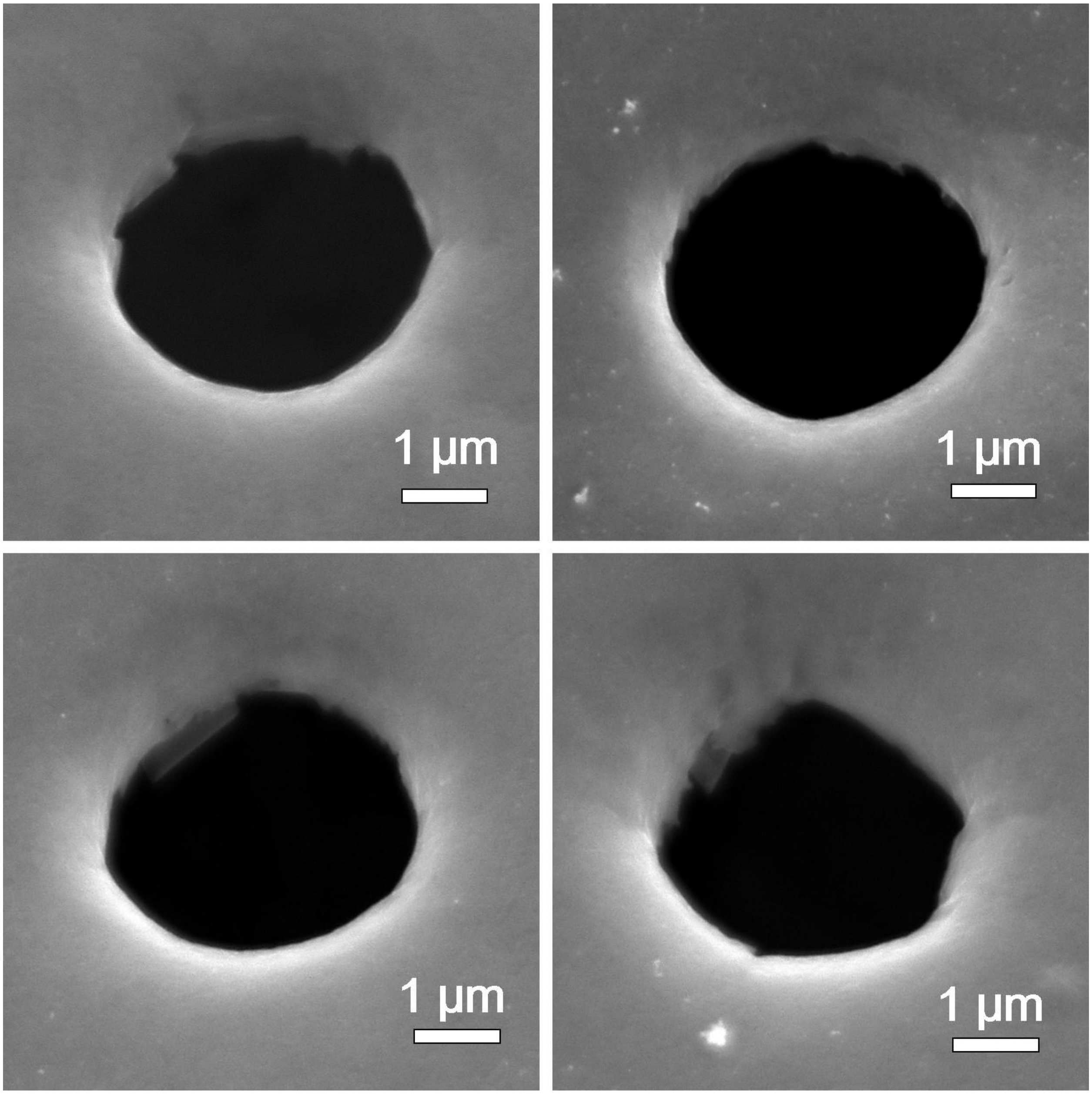

Supplementary Figure 2. A selected collection of SEM micrographs showing the analyzed projectile perforations in UFG Al films.

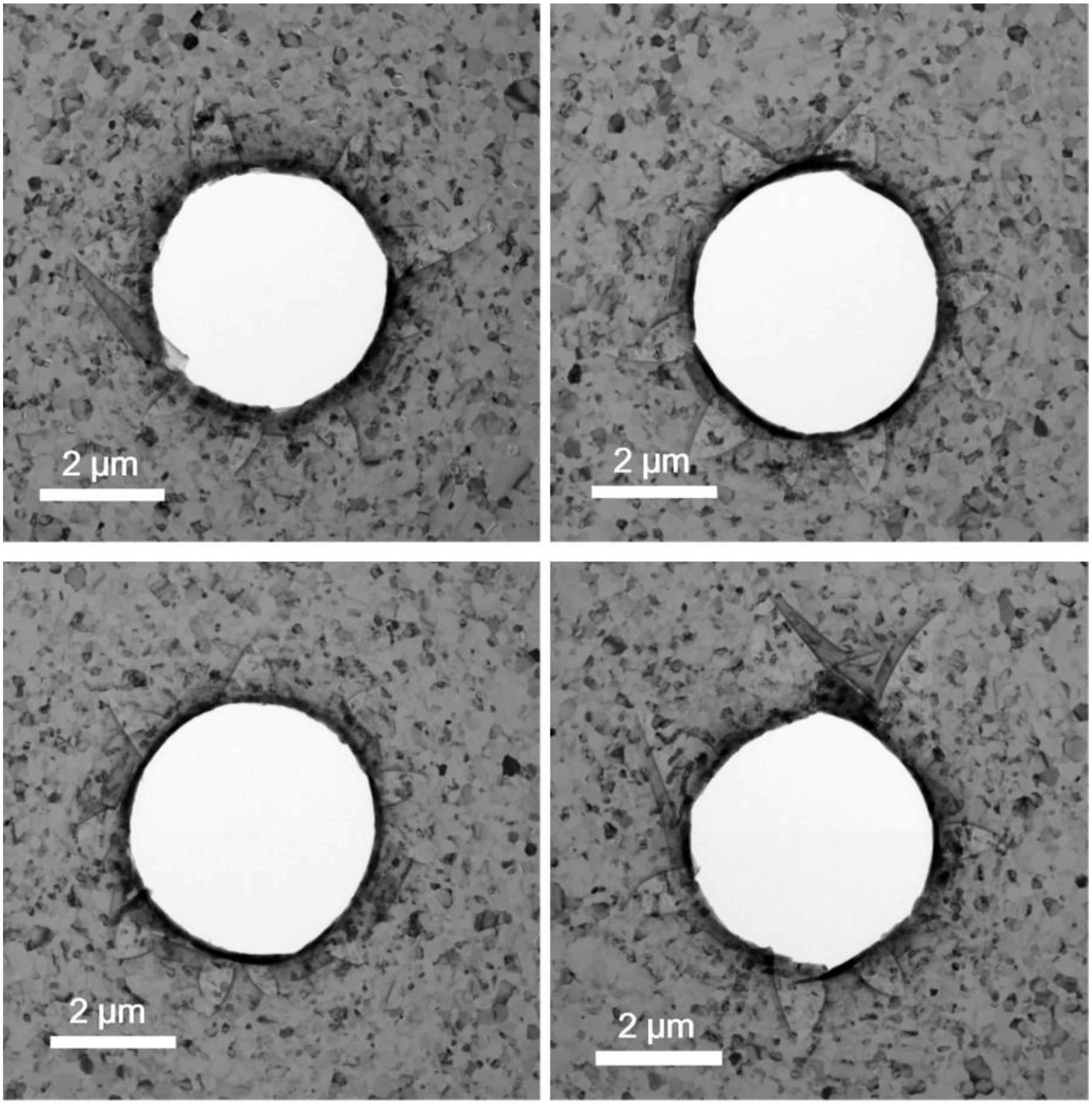

Supplementary Figure 3. TEM micrographs showing projectile penetration induced holes in the impacted UFG Al film. A majority of perforations have nearly circular morphology without obvious cracks.

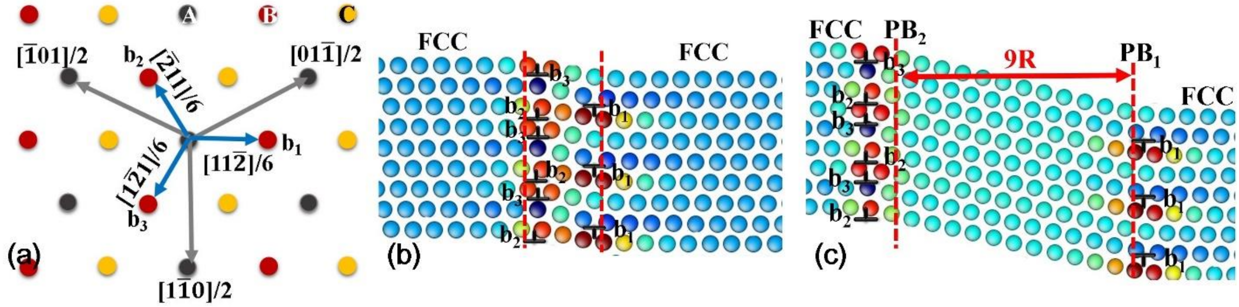

Supplementary Figure 4. Projection views of the (111) plane showing layer-stacking positions for the ...ABCABC... fcc stacking. The stacking can be changed by the glide of any of the three Shockley partial dislocations with Burgers vectors $1/6[11\bar{2}]$, $1/6[\bar{2}11]$ or $1/6[1\bar{2}1]$. (b and c) Relaxed atomic structures of Σ3 {112} ITBs under zero applied stress and under a shear stress of 0.3 GPa. Atoms are colored by common-neighbor analysis. The b_1 partial dislocation arrays are aligned along phase boundary (PB_1) and separate the fcc phase from the 9R phase, while b_2, b_3 partial dislocation arrays form the other phase boundary (PB_2).

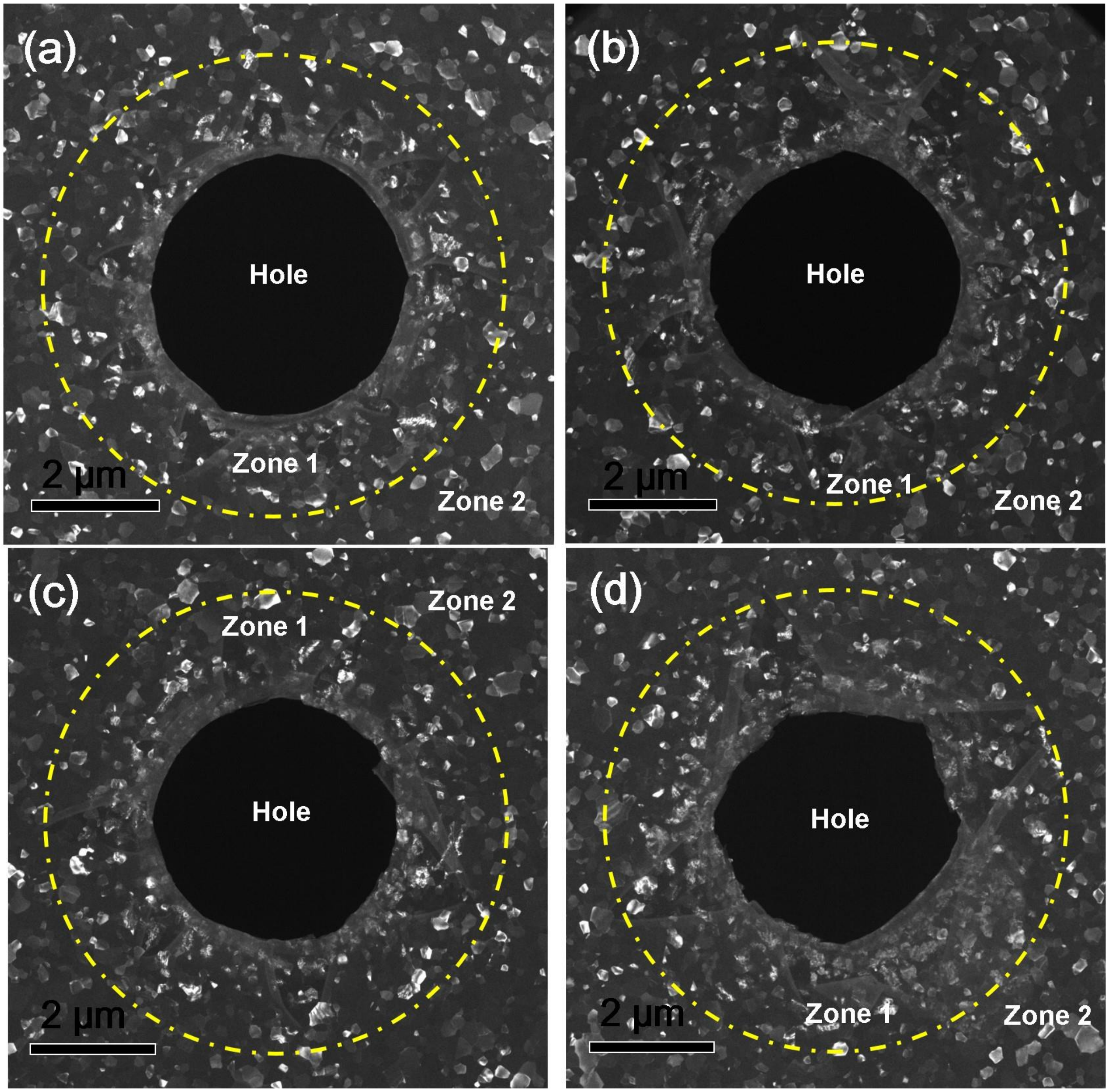

Supplementary Figure 5. Several dark-field TEM images showing the distribution of nanograins near the projectile penetration induced holes ($\vec{g} = <222>$).

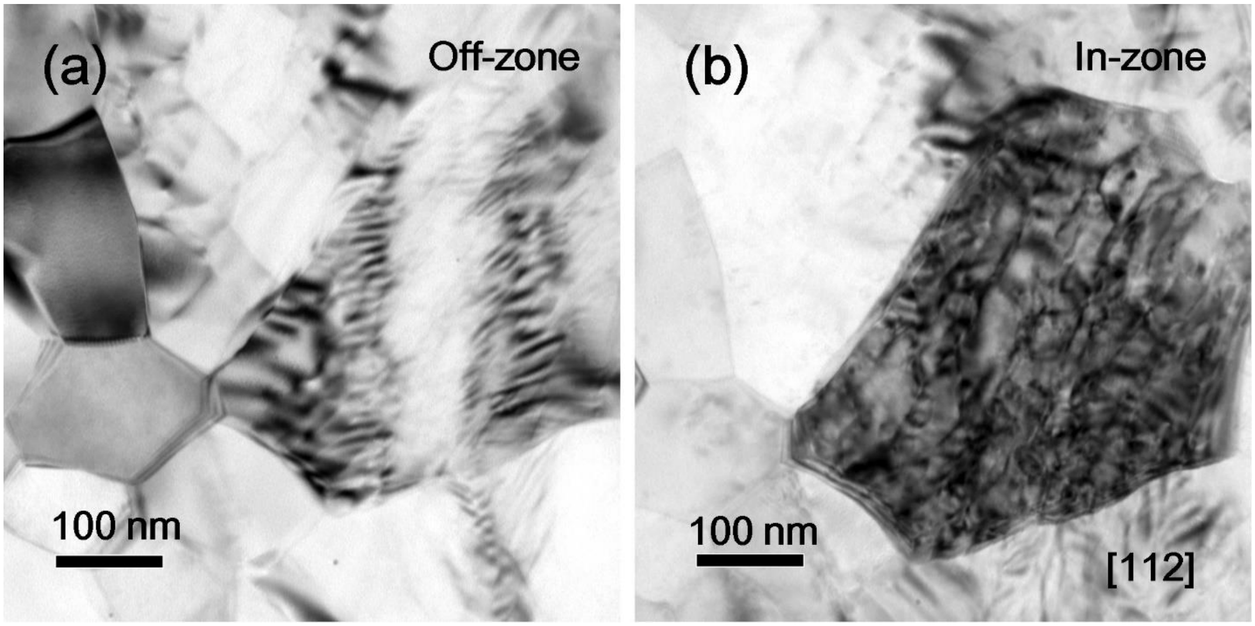

Supplementary Figure 6. TEM tilting experiment to examine the morphology of dislocation networks in grains in zone 1 (highly impacted zone adjacent to the hole). (a) When the large grain is tilted off the <112> zone axis, the dislocation density appears low. (b) In contrast, when titled to the <112> zone axis, a large number of dislocations become visible throughout the grain.

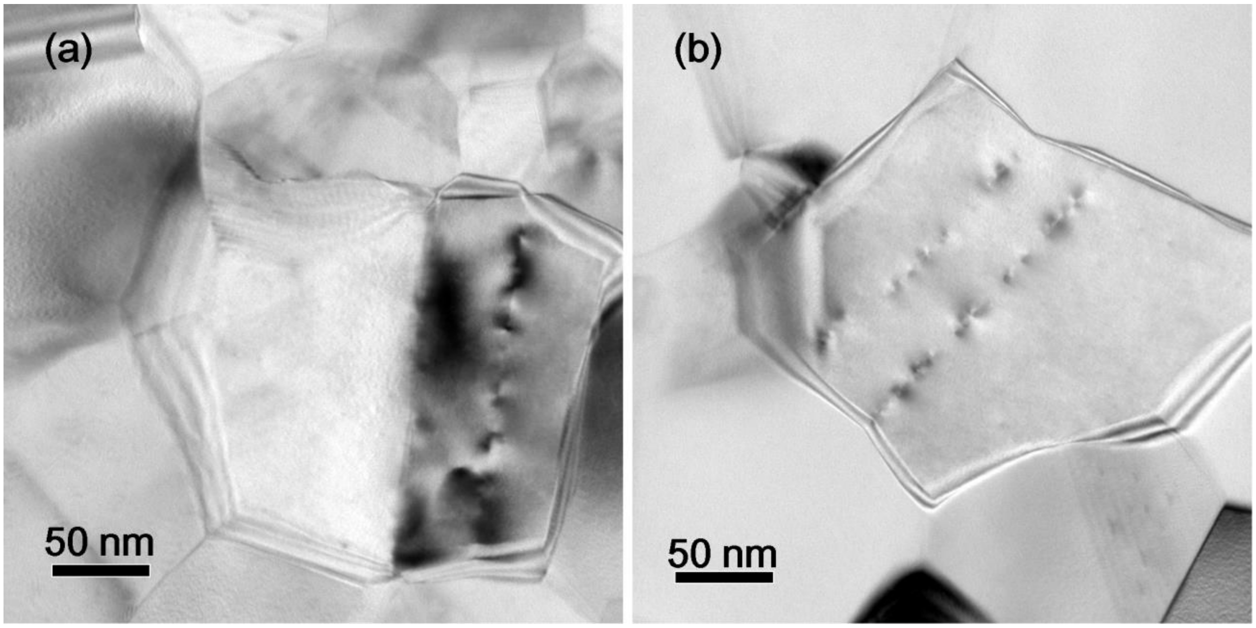

Supplementary Figure 7. TEM images showing the accumulation of dislocations along the preexisting CTBs in grains in zone 2 (the less deformed region, further away from the penetration hole). The CTBs are growth twins introduced during sputtering. The interactions of impact induced dislocations with the CTBs lead to the storage of dislocations (manifested by their cores) along the CTBs. Other than the dislocations along the TBs, the grain interiors have relatively few dislocations.

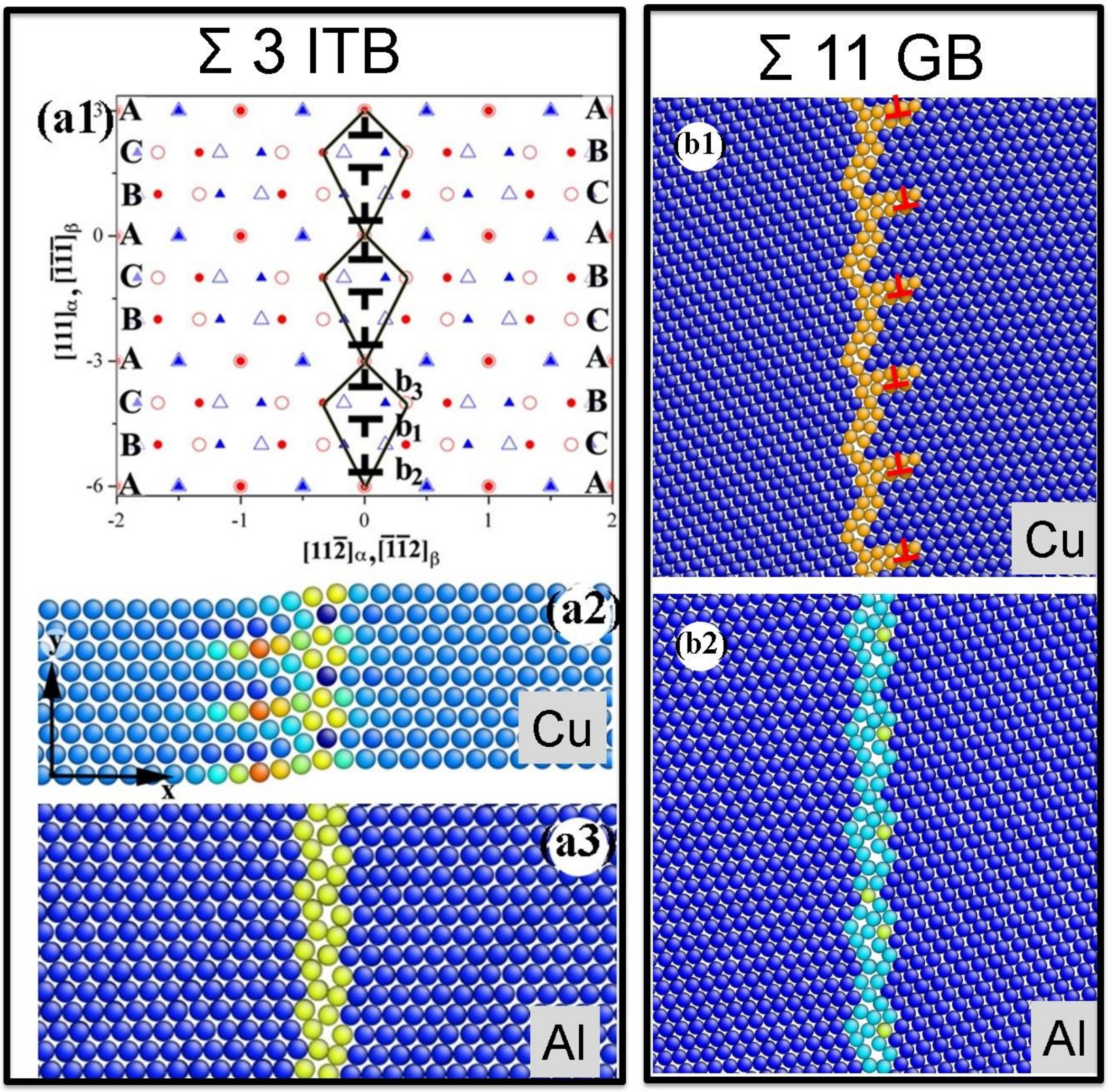

Supplementary Figure 8. Atomic structure of Σ3 ITB and Σ11 GBs in low SFE Cu and high SFE Al. (a1) Dichromatic pattern of a Σ3 ITB, (a2) atomic structure of the Σ3 ITB in Cu, dissociation of one set of partials from ITB due to low SFE, (a3) Atomic structure of compacted Σ3 ITB in Al due to high SFE, and (b1) atomic structure of Σ11 GB in Cu: every seven {111} atomic planes contain one Shockley partial dislocation. All the partial dislocations are dissociated from the GB. (b2) Atomic structure of Σ11 GB in Al. All partial dislocations are constrained inside the GB due to the high SFE of Al.

Supplementary Video 1: Mechanical response of nanoscale columnar Al grains with Σ3{112} incoherent twin boundaries (ITBs) under shock at a speed of 1km/s. The shock direction is inclined to the column boundary at ~ 30 degrees. 9R phase was nucleated at ITBs associated with the emission of pre-existing Shockley partial dislocations at the ITBs. Atoms are colored by common-neighbor analysis. The red atoms represent stacking faults relative to fcc phase (colored in blue).

Supplementary Video 2: Mechanical response of nanoscale columnar Al grains with Σ11 (-252) || (-414) boundaries under shock at a speed of 1km/s. The shock direction is parallel to the column boundary. Shockley partial dislocations were nucleated and emitted at the boundaries. Atoms are colored by common-neighbor analysis. The red atoms represent stacking faults relative to fcc phase (colored in blue).
