# Supplementary material for: High-velocity projectile impact induced 9R phase in ultrafine-grained aluminium
Source: Nat Commun. 2017 Nov 21;8:1653. doi: 10.1038/s41467-017-01729-4 (PMC5698461; doi:10.1038/s41467-017-01729-4)
Supplement: Supplementary file 2 — Description of Additional Supplementary Files [file 41467_2017_1729_MOESM2_ESM.docx]

**Description of Additional Supplementary Files**

File Name: Supplementary Movie 1

Description: Mechanical response of nanoscale columnar Al grains with Ʃ3{112} incoherent twin boundaries (ITBs) under shock at a speed of 1km/s. The shock direction is inclined to the column boundary at ~ 30 degrees. 9R phase was nucleated at ITBs associated with the emission of pre-existing Shockley partial dislocations at the ITBs. Atoms are colored by 2 common-neighbor analysis. The red atoms represent stacking faults relative to fcc phase (colored in blue).

File Name: Supplementary Movie 2

Description: Mechanical response of nanoscale columnar Al grains with Ʃ11 (-252) || (-414) boundaries under shock at a speed of 1km/s. The shock direction is parallel to the column boundary. Shockley partial dislocations were nucleated and emitted at the boundaries. Atoms are colored by common-neighbor analysis. The red atoms represent stacking faults relative to fcc phase (colored in blue).
